# Ethanologenic fermentation by *Parageobacillus thermoglucosidasius* with continuous hot microbubble gas-stripping

**DOI:** 10.1186/s12934-024-02433-x

**Published:** 2024-06-05

**Authors:** Joseph Calverley, Christopher Ibenegbu, Abdulkadir Hussein-Sheik, H. C. Hemaka Bandulasena, David J. Leak

**Affiliations:** 1 Department of Chemical Engineering, Loughborough University, England, LE11 3TU UK; 2 Department of Life Sciences, University of Bath, England, BA2 7AY UK

## Abstract

The increased use of biofuels in place of fossil fuels is one strategy to support the transition to net-zero carbon emissions, particularly in transport applications. However, expansion of the use of 1st generation crops as feedstocks is unsustainable due to the conflict with food use. The use of the lignocellulosic fractions from plants and/or co-products from food production including food wastes could satisfy the demand for biofuels without affecting the use of land and the availability of food, but organisms which can readily ferment all the carbohydrates present in these feedstocks often suffer from more severe bioethanol inhibition effects than yeast. This paper demonstrates the potential of hot gas microbubbles to strip ethanol from a thermophilic fermentation process using *Parageobacillus thermoglucosidasius* TM333, thereby reducing product inhibition and allowing production to continue beyond the nominal toxic ethanol concentrations of ≤ 2% v/v. Using an experimental rig in which cells were grown in fed-batch cultures on sugars derived from waste bread, and the broth continuously cycled through a purpose-built microbubble stripping unit, it was shown that non/low-inhibitory dissolved ethanol concentrations could be maintained throughout, despite reaching productivities equivalent to 4.7% v/v dissolved ethanol. Ethanol recovered in the condensate was at a concentration appropriate for dewatering to be cost effective and not prohibitively energy intensive. This suggests that hot microbubble stripping could be a valuable technology for the continuous production of bioethanol from fermentation processes which suffer from product inhibition before reaching economically viable titres, which is typical of most thermophilic ethanologenic bacteria.

## Introduction

As public awareness of the impact of anthropogenic CO_2_ emissions on climate change grows there is increased urgency to replace fossil fuels in transport applications, which make up a significant portion of global carbon emissions [1]. Lower net carbon emissions associated with biofuels such as bioethanol represent a convenient improvement that can be delivered using current infrastructure and ethanol is already being blended into the existing fuel supply [2]. Bioethanol is mainly produced by the fermentation of sugars, where the most common agricultural substrates used are sucrose from sugar cane and glucose from corn starch, which compete with food production. Increasing production from these sources can lead to direct and indirect land use change, which has the potential to release significant amounts of stored carbon. Therefore, in order to intensify production, it is preferable to consider whole crop utilisation and use of existing agricultural and domestic wastes as feedstocks. When considering alternative feedstocks to simple sugars, it is worth considering whether alternative organisms to *Saccharomyces cerevisiae* bring process advantages, particularly in the use of complex polymeric sugar substrates and sugars other than glucose. Hemicellulose makes up between 25% and 35% of the total biomass (g/g) in wood fibers and up to 40% of non-wood fibers such as grasses (wheat, corn rice) [3], while dry wood has 20–30% hemicellulose [4]. birchwood, rice bran and corn-fiber contain 89.3%, 46%, and 48–54% hemicellulose, respectively [5] which are high percentages of the entire biomass that should not be ignored for economic purposes. In this context, strains of *Geobacillus* spp and *Parageobacillus* spp are of interest because of their ability to hydrolyse and grow on hemicellulose, pectin, mannans and starch. While they are poor cellulose degraders, efforts have been made to add this functionality [6]. The primary reason why *Geobacillus*/*Parageobacillus* spp have not gained a higher profile for bioethanol production is their lower alcohol tolerance compared to *S cerevisiae*. Typically, inhibition starts to be observed at around 2% (v/v) ethanol and concentrations of 4% (v/v) completely inhibit growth [7]. A report by Lynd et al. stated that ethanol concentrations of at least 4% in the broth are required for economic industrial ethanol production [8]. However, it should be noted that, as thermophiles, their optimum growth temperature is close to the boiling point of ethanol so that, during fermentation, some ethanol is naturally removed in the vapour phase. Nevertheless, in order to consider these organisms as viable contenders for bio-ethanol production, ethanol production needs to increase to the equivalent of a batch culture of *S cerevisiae* producing a final concentration of around 8% (v/v) ethanol, a concentration which is unlikely to be achieved by selection of ethanol-tolerant mutants. A more promising strategy would be to enhance the rate of ethanol stripping into the vapour phase.

Dissolved ethanol is stripped from the bulk liquid phase by transfer into gas bubbles passing through the reactor, a process which depends on the bulk phase ethanol concentration, the temperature of both the liquid and gas phase and the contact area between the gas and liquid phases. Modelling this process for typical sparger aerated reactors suggests that, while gas-stripping of ethanol directly from a bioreactor is technically feasible, the volume of gas required per volume of liquid is unrealistic for commercial and practical uses [9]. To make this process more efficient it would be necessary to increase the gas-liquid surface area of the bubbles per unit volume of gas, i.e. to make the bubbles smaller than typically achieved through sparger aeration (typically 0.3–3 mm scale).

Recently, microbubble studies using superheated air for stripping purposes have been demonstrated to have very good mass transfer rates and provided excellent stripping with little heating of the liquid phase [10–16]. Previous studies [17] at elevated liquid temperatures showed good evaporation rates and demonstrate the potential for steady state continuous stripping. Microbubble stripping from a fermentation would remove ethanol from the fermentation medium as it is being produced thus reducing the risk of ethanol inhibition, allowing fermentation of sugars to continue, and the negligible heating of the liquid phase should minimise physiological stress to the microorganism. Using this technology as an *in-* or *ex-situ* ethanol recovery step could reduce the cost of dewatering the product and may remove the inhibitory effects completely [18, 19].

In this study we have investigated the impact of using a side-arm microbubble stripping unit (*ex-situ*) linked to a bioreactor in which the amylolytic thermophile *P.thermoglucosidasius* TM333 was producing ethanol by batch and fed-batch fermentation of hydrolysed waste bread. *P. thermoglucosidasius* TM333 is a strain developed from the ethanologenic mutant TM242 [8], with an enhanced capacity to degrade starch through the addition of an α amylase gene from *G. stearothermophilus* [20]. Although, in the current study, the bread was prehydrolysed to glucose (to simplify the analysis of substrate concentration during the process), using α -amylase produced by the same organism combined with industrial amyloglucosidase, we have recently demonstrated that TM333 has metabolic advantages over yeast for fermentation of bread due to its production of amylase and neopullulanase and the ability to transport and intracellularly hydrolyse the resulting gluco-oligomers [21]. Therefore, it should be straightforward to apply the results of the current study to the fermentation of non-pretreated waste bread, which is a commercially relevant food waste available at significant scale. The results should also translate to fermentation of lignocellulosic substrates using other derivatives of TM242 with enhanced catabolic potential.

A custom-built microbubble stripping unit (MSU), in which a dense, uniform cloud of hot nitrogen microbubbles was produced, was used to strip ethanol from the recirculating fermentation broth with the aim of maintaining ethanol concentrations below toxic levels. Fermentation broth was constantly circulated between the two vessels, while the conditions of the fermentation broth and condensed vapour were recorded over time. The composition of the vapour stream was of interest as concentrated product streams are less energy intensive to dewater.

## Materials and methods

### Design of the microbubble stripping unit (MSU)

**Fig. 1:**
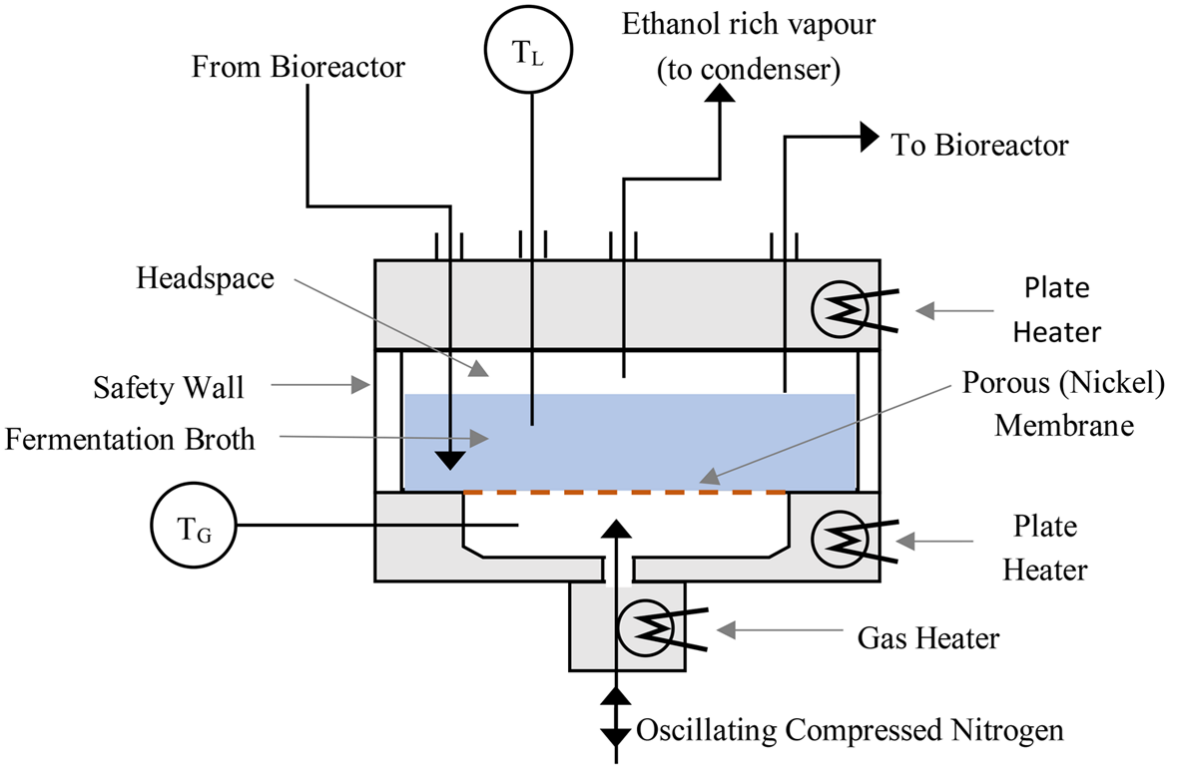
A schematic diagram of the microbubble stripping unit (MSU)

The custom-made microbubble stripping unit used in conjunction with the bioreactor to remove ethanol from the fermentation broth was described by Calverley et al. [17]. Several minor changes to the unit were implemented to make it compatible with this study. A brief description is hereby provided for clarity and a schematic diagram for the MSU is shown in Fig. 1.

The microbubble stripping unit (MSU) was essentially a cylindrical tank to contain the circulating fermentation medium, with a microbubble sparger at the base of the unit. In order to reduce condensation occurring on the vessel walls, the height of the glass cylinder was chosen carefully such that the liquid level used in the experiments (25 mm) left only a small section of glass surface exposed in the headspace of the unit (~ 2 mm once the cylinder was clamped in place). The total glass cylinder height was 35 mm and had an internal diameter of 140 mm (borosilicate glass, ScottGlass Ltd.). The glass cylinder rested on a groove on the base of the unit to form a sealed container when the lid of the MSU was bolted in place. To withstand high gas temperatures at the inlet and the autoclaving procedure, aluminium was chosen as the material for the base and the lid. In order to improve the chemical resistance of the unit, both the base and the lid were anodised and PTFE dipped (SPL Blacking, Loughborough).

A dense cloud of microbubbles was generated by an oscillatory gas flow produced using a laboratory scale fluidic oscillator (Perlemax Ltd., Sheffield), with a gas flow rate of 2 SLPM (standard litre per minute, 273.15 K, 10^5^ Pa) controlled by a mass flow controller (Alicat, MCR-series). The sparger at the bottom of the tank was made with a porous nickel membrane (Micropore Technologies Ltd, Redcar, UK.) which has an average pore size of 20 μm and a hexagonal pitch of 180 μm. The nickel membrane was cut to a 100 mm diameter circle and placed in a clamping ring, to provide a good seal. The clamping ring slotted into a groove ground into the base of the unit. The membranes were cleaned thoroughly before experiments, in a two-stage process. First, the membrane was placed in a sonication bath containing 2 M citric acid (Fisher Scientific) for 15 min. Secondly, the membrane was placed in a sonication bath containing 4 M sodium hydroxide (Fisher Scientific) for 15 min. Finally, the membrane was washed with sterile distilled water prior to use. The average bubble size could not be measured optically at the appropriate conditions as the fermentation liquid was opaque, thus the bubble size was assumed to be the same as in our previous studies [17].

One of the alterations to the design of the MSU was to move the gas heater to the base of the unit. Using the overall gas flow rates in our previous (~ 15 SLPM, 273.15 K, 10^5^ Pa) studies would have been wasteful of the compressed nitrogen, and as such a much lower flow rate was used in this work. Therefore, to reduce the residence time of the heated gas, the gas heater was integrated into the base of the MSU for the current study. This consisted of a hollow aluminium cylinder, to allow the gas to flow through with negligible resistance. The whole cylinder was heated using two cartridge heaters (100 V, RS-PRO) controlled by an in-house built on/off control unit, which monitored the temperature of the block using an RTD (Pt100).

The temperature of the liquid inside the MSU was maintained at 60 °C to reduce the thermal shock to the microorganisms being circulated along with the fermentation liquid. This was achieved by heating the entire base of the unit to 65 °C using two cartridge heaters (120 V, RS-PRO). The cartridge heaters were controlled by an in-house built on/off controller, which measured the temperature of the liquid using an RTD (Pt100). The liquid temperature was measured using a data logger (CompactDAQ, National instruments) using a thermocouple (K-type, RS-Pro). The thermocouple was placed within the unit so that it was approximately half the liquid height from the base of the unit. If the liquid temperature deviated more than 2 K from the desired temperature, the temperature set-point of the base plate was adjusted to compensate. To reduce condensation on the lid surfaces, the top plate of the unit was heated to 75° C in the same manner as the base plate.

### Bioreactor operation

The bioreactor used was a 5 L double walled glass vessel and the operating conditions were maintained using a Biostat B-plus controller (Sartorious Stedim Biotech). The temperature of the bioreactor was maintained at 60° C by supplying hot water into the outer jacket of the vessel. The temperature of the fermentation broth was measured using an RTD (Pt100), while the dissolved oxygen concentration (pO_2_) was measured as percent of saturation using an OxyFerm FDA (Hamilton) probe which was calibrated at 60° C just before starting the experiment. The pH of the fermentation broth was measured with a probe (EasyFerm Plus, Hamilton) and controlled to pH 7.0 using a PID control system with activating pumps supplying either 4 M phosphoric acid (Merck) or 4 M potassium hydroxide (Fisher Scientific). Filter-sterilised compressed air was introduced through a ring-sparger below the lowest of two Rushton turbine impellers on the agitation shaft. The in and out media flow rate was controlled by the Biostat feed pump (previously calibrated) and a tube from the off-gas of the bioreactor was connected to one end of a Dreschel bottle containing 500 mL of deionised water (12 mΩ). A further tube was connected from the other end of the bottle to a second Dreschel bottle containing 15 mL of water, with both bottles cooled on ice to trap the vapour stripped from the bioreactor. The volumes of these bottles were checked at various intervals, and small samples of this liquid were taken for ethanol quantification. The bioreactor was operated between 4 and 5 L final working volume to account for the extra volume required for the additional feed and was connected to the MSU via two flexible tubes for the circulation of the fermentation broth.

The assembled bioreactor containing 2.5 L deionised water (12 mΩ) was sterilised by autoclaving at 121° C for 15 min (monitored using a probe in a dummy vessel). Then, the water in the vessel was pumped out through tubes connected to the sampling port replaced with 2 L of sterile fermentation broth containing 5 g/L NaCl, 16 g/L peptone, 8 g/L yeast extract (2SPY media) and 40 g/L glucose made from waste bread in deionised water (DI) as described [21]. Briefly, waste bread from UK commercial sandwich production (25% ww dried and milled) was gelatinised (100˚C) and liquefied (85˚C) with *P. thermoglucosidasius* strain TM333 alpha amylase at pH 6.5 and subsequently saccharified to glucose with amyloglucosidase (Novozymes, Denmark) at pH 5.5 and 60˚ C for 3 h. This medium was then concentrated in an Edwards Modulyo EF4 freeze dryer (Edwards High Vacuum, UK), diluted to 250 g/L glucose or 40 g/L in 2SPY and warmed in a water bath to 50˚C before filter sterilization in 1 L 0.2 μm filter cups (Fisher Scientific, UK) under vacuum. Antifoam 204 (Merck) was added at 1% for foam control and the pH of the 2 L waste bread sugar medium was adjusted to 7.0 with 4 M NaOH in DI water, while that of the 250 g/L sugars (to be used as feed) was adjusted to pH 4 (to avoid microbial contamination) with 4M H_3_PO_4_.

*Parageobacillus thermoglucosidasius* TM333 (provided by ReBio Ltd, Surrey, UK), stored at -80° C in 18% glycerol, was inoculated on to 5 tryptone soy agar (TSA, Sigma) plates and incubated overnight at 60° C. Then, 4 × 150 mL of tryptone soya broth (TSB, Sigma) in 500 mL baffled shake flasks were inoculated with cells from the 5 agar plates, and incubated in a shaking incubator (Innova 44) for 4 h at 60˚ C and 200 rpm. This culture (500 mL) was subsequently used to inoculate the bioreactor and aerobic conditions were maintained for approximately 3 h (agitation rate of 600 rpm and air flow rate of 1 vvm) to increase the cell density. Subsequently, agitation and aeration were reduced to 300 rpm, and 0.2 vvm respectively to initiate fermentation. Liquid circulation between the bioreactor and the preheated MSU was activated at this stage. After 10 h, 300 ml of the concentrated sugar solution (250 g/L) of waste bread glucose in 2SPY medium was fed into the bioreactor and this feed was also used for the subsequent fed-batch stages.

To monitor the growth of the microorganism in the bioreactor, samples were taken at intervals and the absorbance at 600 nm (OD_600_) measured using a spectrophotometer (Jenway 6305). Some of each sample was centrifuged at 3200 x g for 15 min and filtered through a 0.2 μm nylon filter (Phenomenex, USA). The filtered medium was frozen for later analysis of sugars and ethanol by HPLC and gas chromatography.

### Process integration

**Fig. 2:**
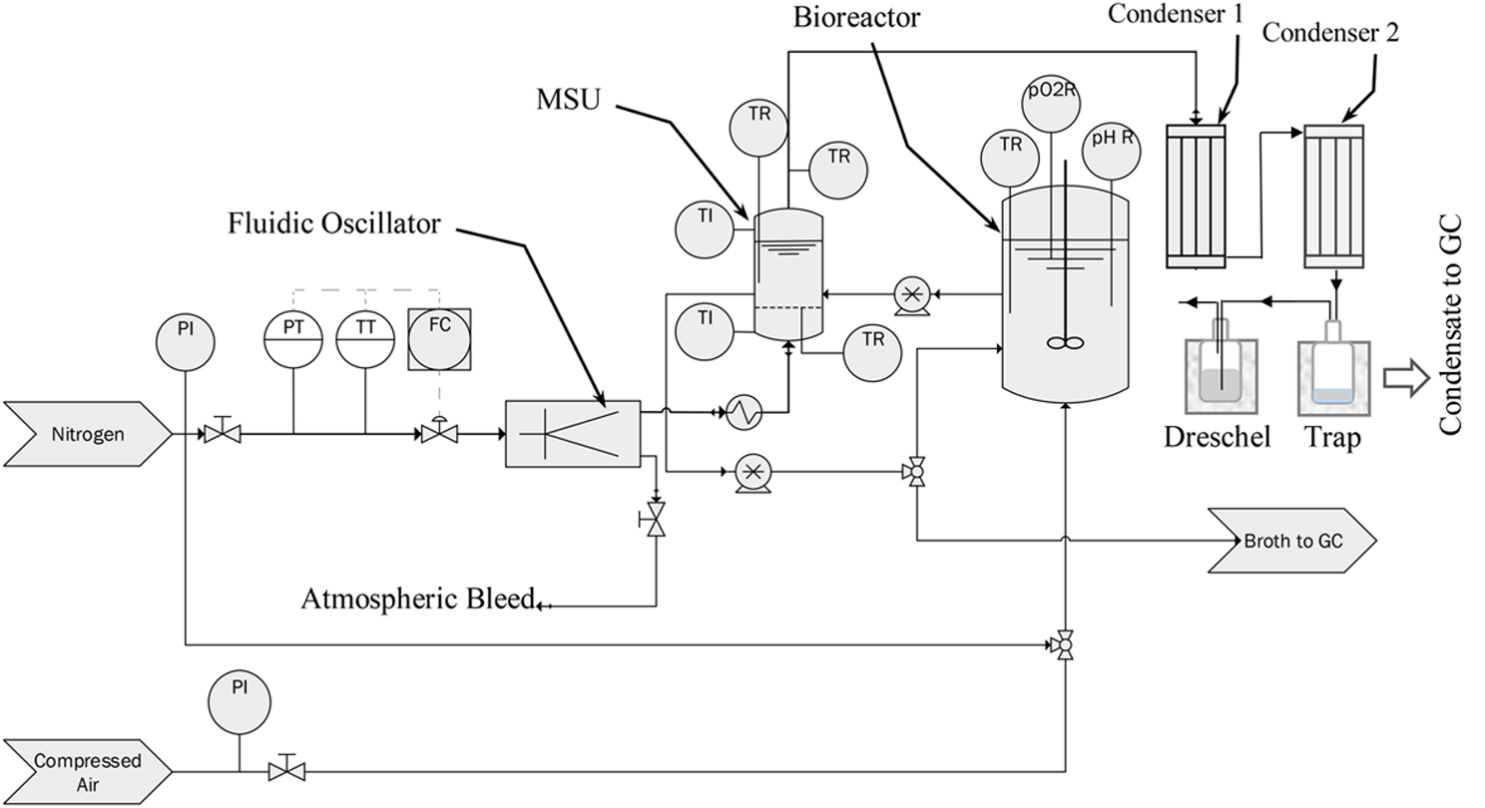
Simplified Process Flow Diagram (PFD) for the fermentation system

Figure 2 shows the experimental setup of the system. The fermentation broth was continuously recirculated between the bioreactor and the MSU using two peristaltic pumps at a flow rate of approximately 335 mL/min. The volume of liquid resident in the MSU at any time was 425 mL (liquid height of ~ 25 mm), giving a residence time of ~ 1.27 min. (This was a measured value from a non-gassed system, so the total volume would have been slightly higher during operation, due to the presence of entrained gas bubbles). The broth entered the MSU from the bioreactor through a stainless-steel tube (Swagelok, 6 mm) running through the lid of the MSU and was released below the liquid level (~ 10 mm from the base of the unit) to reduce foaming. The liquid left the MSU through another flexible tube passing through the lid of the unit which was positioned 25 mm from the base of the unit and re-entered the bioreactor. Placing the outlet tube at the liquid level required (25 mm) and setting a slightly higher pumping rate for the outlet tube compared to the inlet allowed maintenance of a constant predetermined liquid level in the MSU.

In order to maintain fermentative conditions inside the system, the stripping gas used in the MSU was nitrogen (BOC). The feedback loop on the fluidic oscillator was 5 m long, which resulted in an oscillation frequency of 323 Hz, measured using an oscilloscope (Picoscope, 6402 C) and data processed using MATLAB (2015b). One half of the oscillating flow was bled to the atmosphere, as the fluidic oscillator has two outlet ports, while the other half was passed through an electrical heater at the base of the MSU to reach a gas inlet temperature of 75 °C. The gas heater was set to 350 °C as measured by an RTD (Pt100) feeding into the temperature control unit that controlled the temperature of the aluminium base and lid of the MSU.

The ethanolic vapours exiting the MSU were condensed by using two glass condensers (Quickfit) in series cooled with diluted (~ 50% [v/v]) automobile antifreeze concentrate (Halfords). This was maintained at -15° C by a refrigeration unit (LABPLANT, PB-80/2 Refrigeration Bath, UK). A bottle (Bottle A) with an exit connector chilled on ice was directly connected to the lower condenser for collection of ethanol extracted by the MSU. At intervals, the condensate in bottle A was collected by decantating into a graduated measuring cylinder, the volume measured, 0.5 mL sample taken for analysis and the remainder saved in a Durham collation bottle. Subsequent samples from the condenser were also measured and added to the same, capped, collation bottle. A tube from the gas outlet of bottle A was connected to a Dreschel bottle (Bottle B) containing deionised water (200 cm^3^, 12 mΩ) also cooled on ice, which acted as a liquid trap to trap any traces of ethanol in vapour being gassed out from bottle A. The volume of this liquid trap was also measured at intervals by decanting into a graduated cylinder, then returning to the original bottle, with 0.5 mL samples being kept for ethanol quantification, i.e. none of the liquid (apart from analytical samples) was removed from bottle B for collation.

The heating control units that control the temperatures of the top and base aluminium plates and the gas heater were turned on one hour before the liquid circulation was initiated. To avoid dampening of the oscillating flow, the inlet nitrogen gas steam was not filtered; it was assumed that the stream was thermally sterilised by the gas heater (temperature of 350° C).

### GC and HPLC

Gas chromatography was used for the measurement of ethanol concentration in both the filtered broth and condensate samples. 1 mL of each diluted sample was combined in chromatography vials with the internal standards appropriate to each sample, 100 µL of the low concentration standard (40% [v/v] propanol (Fisher Scientific) in water) for the fermentation broth or 200 µL of the high concentration standard (80% [v/v] propanol in water) for the condensate. Each sample and internal standard mixture was injected (1 µL, 1:50 split ratio) into a gas chromatograph (Agilent 7890 A), containing a J&W DB-WAX column (Agilent Technologies, 30 m x 0.25 mm with 0.25 μm coating) with an injection temperature of 150° C and at an oven temperature of 45° C with a 1 mL/min helium mobile phase. Each sample was injected five times and the average ratio used to calculate the concentration by comparison with standard solutions of ethanol in water. For the fermentation broth composition, standard solutions of 5% (v/v) and 2.5% (v/v) of ethanol in water were used, while standard solutions of 20% (v/v), 10% (v/v) and 5% (v/v) were used for the condensate composition.

Sugars, ethanol and other acids/metabolites were subsequently analysed using an Agilent 1100 HPLC system as previously described [21] with a Phenomenex ROA organic acid H^+^ column 300 × 7.8 mm (Phenomenex, USA), 5mM H_2_SO_4_ as eluent, column temperature of 65˚ C, flow rate of 0.6 mL/min and 20µL injection.

## Results and discussion

**Fig. 3:**
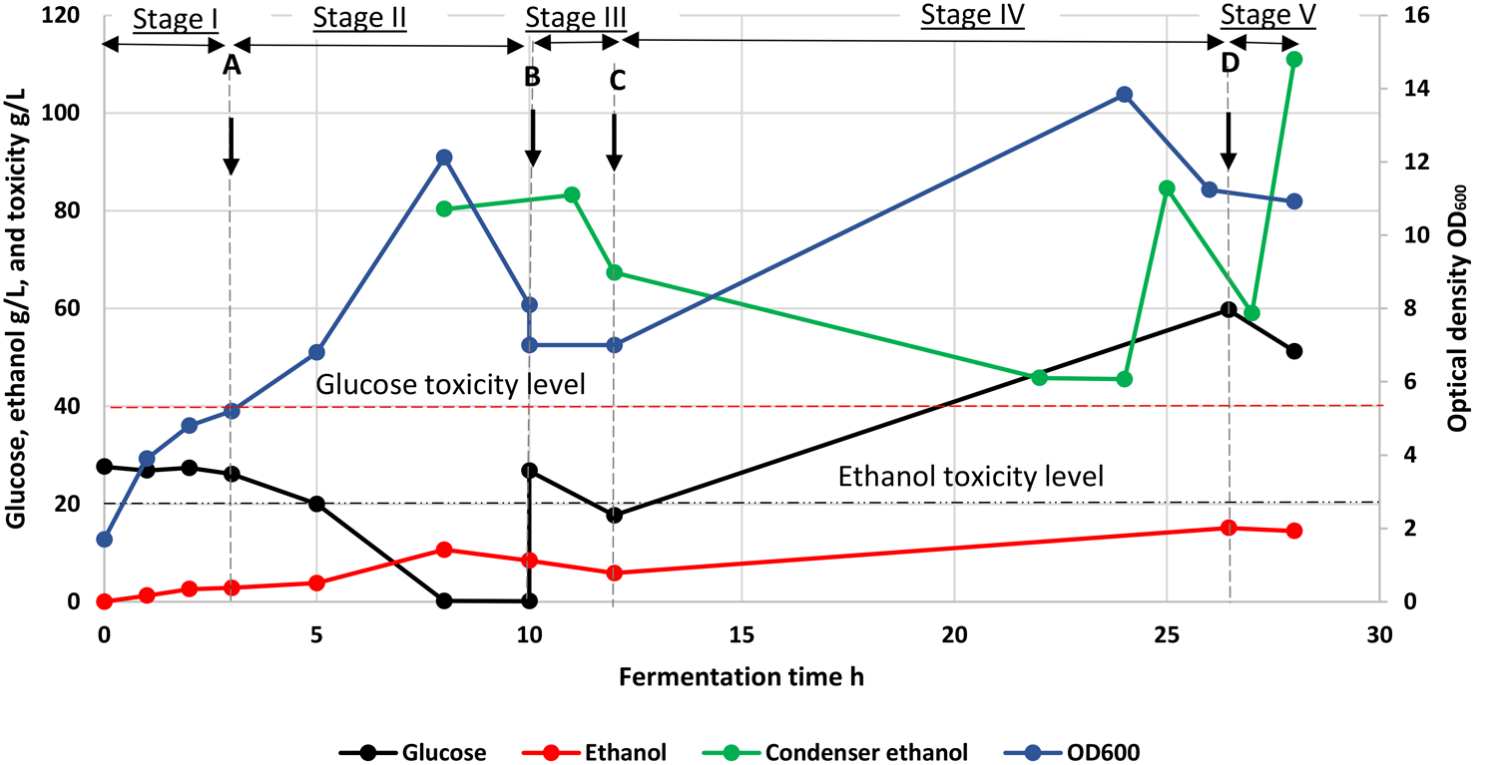
Fermentation of waste-bread feed with *ex-situ* microbubble stripping; Stage I (aerobic batch), Stage II (batch fermentation), stage III (extended batch, fermentative), stage IV and V (fed batch 1 & 2 fermentative). **A**: aerobic to anaerobic switch, **B**: batch feed addition; **C**: start of fed batch 1 fermentative stage, 1.8 mL/min; **D**: start of fed batch 2 fermentative stage, 2.8 mL/min

The time course for the ethanol fermentation process showing residual glucose concentration, ethanol concentration in the reactor, cell density measured as OD_600_ and condenser trap bottle (A) ethanol concentration is shown in Fig. 3.

The fermentation process can be separated into various stages. Stage I (hr 0–3) was the aerobic batch growth stage, in which aeration at 1vvm with 600 rpm agitation was used to achieve rapid cell growth. Stage II (hr 3–10) was the batch fermentation stage, in which the aeration and agitation rates were reduced to 0.2 vvm and 300 rpm respectively, and MSU circulation was turned on with the stripping process activated. Stage III (hr 10–12) was an extended batch fermentation stage, where 300 mL of concentrated feed solution was added as a single aliquot. Stage IV (hrs 12 to 26.45) and V (hr 26.45-28) were the fed-batch 1 and 2 fermentative stages where the concentrated sugar medium feeding rates were 1.8mL/min and 2.8 mL/min, respectively. The fermentation was terminated after 28.0 h. The variation in key parameters are discussed below, by stage.

### Stage I: aerobic stage (0–3 h)

The OD_600_ is an approximate measure of the cell concentration in the fermentation broth. Therefore, in a simple batch culture the health of the culture, can be inferred from changes in OD_600_. Over the aerobic growth stage where the broth was aerated with 2.5 L/min of air, the primary aim was to encourage cell growth to build a robust culture for rapid production of ethanol. During this stage, the OD_600_ increased to approximately three times the initial value, consistent with other studies using *P. thermoglucosidasius* [22, 23]. A low concentration of ethanol was produced during this stage because the microorganism is Crabtree-negative [24] and does not express fermentative alcohol dehydrogenase in an oxygen rich environment [25]. The liquid recirculation with the MSU was not operational during the aerobic stage; therefore, no data for the ethanol concentration in the condensate was collected.

### Stage II: batch fermentation stage (3–10 h)

**Fig. 4:**
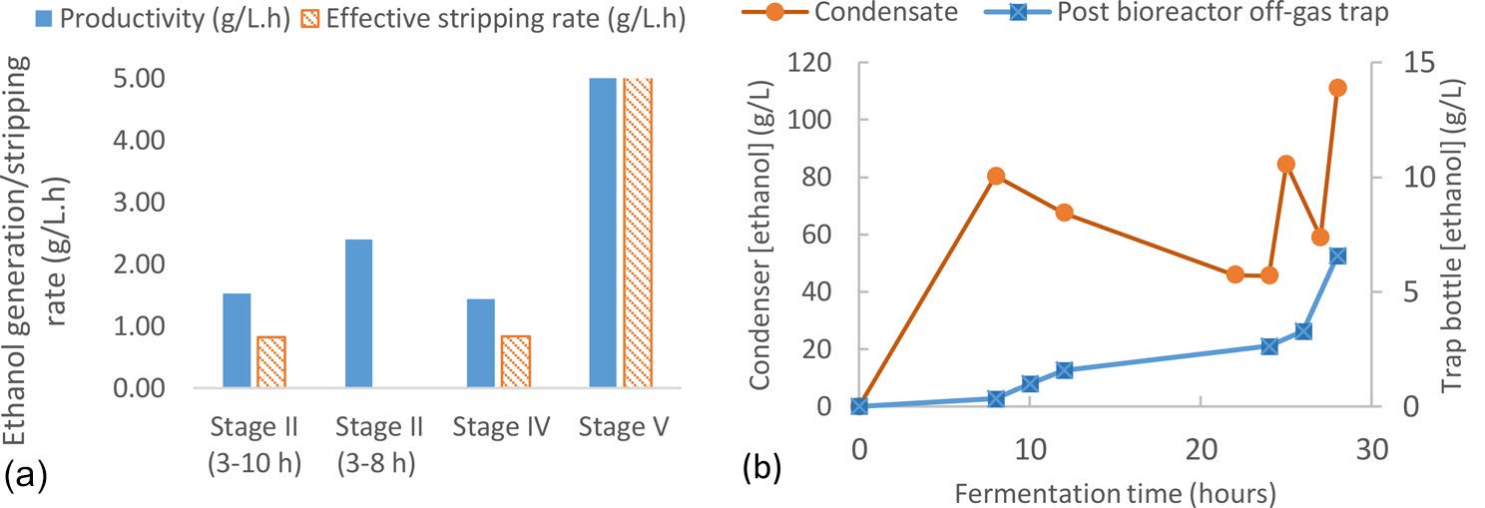
(**a**) Ethanol productivity and effective stripping rate by stage calculated based on sugar consumption, assuming a yield of 90% of the theoretical maximum. As glucose was exhausted after 8 h both the average (3–10 h) and actual (3–8 h) productivity are presented for stage II. Stripping rate could only be estimated over the period, 3–10 h. (**b**) ethanol concentration of individually collected samples from the MSU condenser trap (bottle A) and from the bioreactor off-gas (note that bottle A was emptied into a chilled storage collation bottle after each measurement to avoid losses due to constant gas flow, whereas the concentration in the off-gas was accumulative)

During the batch fermentation stage (Stage II), the aeration rate in the fermenter was reduced to 0.2 vvm, and the agitation rate was reduced to 300 rpm to maintain microaerophilic conditions (a small amount of oxygen is required to allow fermentative growth in this medium), while the liquid circulation between the MSU and fermenter was started. The OD_600_ continued to increase up to ~ 8 h and then decreased over the next 2 h due to carbon source exhaustion, and further at 12 h due to the dilution effect of the batch feed addition at 10 h (Fig. 3). Post fermentation analysis showed that the glucose in the bioreactor had been exhausted by 8 h so the subsequent reduction in OD_600_ might also reflect a degree of cell lysis which is typically observed after rapid sugar starvation of *P thermoglucosidasius* NCIMB 11955 (unpublished observations, also see 22). The initial increase in cell density could also partially reflect the reduction in liquid volume in the system due to evaporation from the MSU. Over 95% of the maximum theoretical expected ethanol (at 90% assumed yield) at stage II was recovered and collected in bottles (Table 1; Fig. 4). This stage demonstrates that hot microbubble stripping had no evident harmful effects on cells recirculating in the fermentation broth, complementing microbubble research in biological systems under isothermal conditions [26].

The ethanol concentration in the bioreactor increased from 2.8 to 10.6 g/L during the 3–8 h period (Fig. 3) which allows estimation of an approximate stripping rate. From the measured glucose utilisation rate (average 5.22 g/L.h) and assuming an ethanol yield of 90% of the theoretical maximum gives an ethanol productivity of 2.40 g/L.h (Fig. 4). Based on the total volume within the system and allowing for the ethanol remaining in the bioreactor gives an effective stripping rate of 0.83 g/L.h for stage II (based on the actual recovery, or 0.88 g/L.h based on assumed yields), which is significantly less than the stripping rates produced in our previous study with pure ethanol-water mixtures (~ 10–20 g/L.h) [27]. In addition to the low concentration driving force for mass transfer, this result is likely due to a combination of several factors. The maximum stripping gas temperature used in this study was 75° C compared to 120° C in our previous work. Additionally, the antifoam agents, salts, cell secretions, waste bread medium etc. all affect the mass transfer rate by altering the conditions at the gas-liquid interface [28, 29].

The concentration of ethanol in the first recovered condensate (at 8 h) was high in comparison to the subsequent values (12–24 h), especially considering the low concentration of ethanol in the broth (Fig. 3). This is consistent with previous work [17] and is likely due to the heating of the condenser from the hot, humid outlet of the MSU to the condenser’s steady-state temperature [17]. After this point, and continuing into the start of the fed-batch fermentation stage, the decrease in concentration is due to the decrease in liquid ethanol concentration, as this is a key parameter in the determination of the driving force for mass transfer [30].

### Stage III: extended batch fermentative stage (10–12 h)

At 10 h, a 300 mL aliquot of concentrated waste bread sugar medium was added which increased the sugar content in the reactor to 26.8 g/L. Fermentation of these additional sugars was continued until 12 h when the sugar concentration had decreased to 17.7 g/L. It was also observed that the OD_600_ dropped from 8.1 at 10 h to 7.1 at 12 h, while ethanol in the fermentation reactor decreased from 8.4 to 5.8 g/L. (Fig. 3) over the same period. The drop in OD can be explained mainly from the effect of dilution by the feed addition, but the reduction in ethanol concentration suggests that the rate of removal exceeded that of production. As the cells had accidentally been starved of sugars at the end of previous stage, it is likely that the surviving cells were gradually recovering and their lower specific sugar consumption rate might have allowed for partially aerobic growth; therefore, this stage would be expected to produce a lower yield of ethanol and was too short to generate meaningful data.

### Stage IV and V: fed-batch 1 and 2 stages (12–28 h)

At 12 h, a continuous concentrated waste bread sugar medium feed at 1.8 mL/min was initiated marking the transition to the fed-batch fermentation stage 1, which ran overnight with no sampling and data recording. The lack of regular decanting of bottle B during this period could have resulted in considerable ethanol losses. It is notable that the glucose data measured after 12 h suggested that the cells were recovering from the brief glucose starvation period and, by 24 h, the cell density had increased (note that this was now in a larger volume so not strictly comparable to the value after 8 h). However, the glucose concentration in the culture had increased, indicating that glucose supply was exceeding demand, and by 24 h the concentration exceeded the known toxicity limits for *P. thermoglucosidasius*, above which the maximum growth rate reduces. Based on the sugars metabolised, at 26 h approximately 54% of the expected ethanol was recovered after removal by the ethanol stripping system. This assumes that cells had returned to fully fermentative growth by 12 h.

From the previous analysis of metabolic rates that could be supported by ethanol stripping in this system it was possible that the glucose feed rate of 27 g/L.h exceeded the gas-stripping capacity (although it should be noted that this will increase with an increase in ethanol concentration in the reactor). However, after 24 h of culture the ethanol concentration was still below inhibitory levels in the bioreactor despite glucose accumulating. The fact that the ethanol concentration in the condenser bottle after 12 h was relatively low, suggests that production was limited by the effects of the earlier glucose starvation combined with subsequent glucose toxicity, rather than limited stripping and subsequent ethanol toxicity. Nevertheless, it is notable that the glucose concentration at 28 h was lower than that at 24 h suggesting that the spike in glucose concentrations had been transient, and was recovering by 26.5 h. Notably, this was accompanied by an increase in ethanol concentration in the reactor (Fig. 3) and increases in ethanol stripping (Fig. 4). Despite the circuitous route to get to this point, the data points between 24 and 28 h act to demonstrate the effectiveness of *ex-situ* gas-stripping for increasing the productivity of ethanol. Cells were growing at a high cell density with a high production rate of ethanol, as witnessed by the concentrations in the condensate bottles. Yet the ethanol concentration in the culture was below the levels which start to affect the growth of *P thermoglucosidasius*. The ethanol productivity at stage IV was 1.43 g/L.h, while the effective stripping rate of ethanol was 0.83 g/L.h. These values were affected by the lack of sample collection overnight leading to ethanol losses, which were unaccounted for. Additionally, they probably represent an average value of continued low production after 12 h, followed by higher production and stripping after a return to full fermentative metabolism. Over the period 25 to 28 h the ethanol concentration in the condensate bottle A was very high, ranging between 46 and 111 g/L, and was therefore, in some cases, more concentrated than the product stream of a traditional fermentation process (87–95 g/L) using baker’s yeast [28, 31].

At 26.45 h (start of fed batch 2 fermentative stage) the feeding rate was increased from 1.8 mL/min to 2.8 ml/min (56% increase in feeding rate). Interestingly, ethanol productivity remained relatively high and the glucose concentration in the bioreactor fell, suggesting that the culture may have adapted to tolerate the high glucose concentration, a phenomenon which has been reported elsewhere [7]. However, little useful data on the limits of ethanol extraction was obtained beyond this point, so the experiment was terminated soon afterwards. The ethanol productivity for stage V was 4.72 g/L.h while the ethanol stripping rate was 4.80 g/L.h, indicating that the post-starvation recovery and possible adaptation of the bacterial cells was complete, and they were fermenting rapidly. Considering the starting and added sugars during the entire fermentation, we would have expected the ethanol concentration to increase in the reactor and collection vessels to a maximum of 8.24% v/v if all the broth and feed glucose was metabolised (7.42%, if 90% of maximum theoretical).

### Substrate mass balance

Based on the measured volumes of all the constituent components of the system, a sugar mass balance was performed to evaluate the overall productivity of the system. It was assumed that ethanol yield from the fermentation was 90% of the theoretical maximum, a yield typical of industrial bioethanol yeasts from glucose [30]. The overall volume (starting volume with fed broth and removal accounted for) at the end of the experiment, was 4.57 L, with the microbubble removal of an accounted amount of 0.43 L ethanolic liquids collected in bottles, thereby leaving behind an assumed 4.14 L in the reactor. It must be noted that there would have been some additional liquid losses due to evaporation, which are not accounted for in this mass balance, especially with Table 1 showing that the total ethanol accounted for after 28 h was only around 51% of that expected when all the added, remaining and consumed sugars had been considered (assuming 90% of the theoretical maximum ethanol yield). This implies an unaccounted “loss” of around 49% of the ethanol, assuming all sugars were converted to alcohol. The total amount of sugar left at the end of fermentation out of a total of 583 g was 212 g, which represents a glucose consumption of 371 g (Table 1). Assuming a yield of 90% of the theoretical maximum based on consumed sugars, this would therefore have produced 170 g of ethanol, which over the entire final volume was equivalent to an overall ethanol concentration of 4.72% (v/v, 37.24 g/L) without stripping, with an overall ethanol productivity of 1.49 g/L.h, taking into account that the start point was at 3 h as this was when the process was switched to fermentative conditions. In comparison, if the initial sugar at 3 h (26.12 g/L, in 2.5 L) was consumed in a batch process by 8 h, the ethanol concentration would have been 1.52% (v/v), corresponding to an overall ethanol productivity of 2.40 g/L.h. The final stage (stage V) of fed-batch fermentation with microbubble stripping showed a consumption and production rate above that which would be possible over a complete batch process and is therefore a significant process improvement. The range of microorganisms tabulated by Azhar et al. [31] had ethanol productivities in the range of 0.17–1.38 g/L.h for batch cultures and the fed-batch process had an overall ethanol productivity of 3.46 g/L.h using a wild strain of *S. cerevisiae*. Elsewhere, a fermentation with gas stripping process using pretreated sugarcane bagasse as substrate and *Kluyveromyces* sp. IIPE453 as production organism had a maximum ethanol generation rate of 1.25 g/L.h [32].), Thus, the ethanol generation in both stages II and IV were operating at rates competitive with or higher than other microorganisms. The ethanol recovered at each sampling stage is shown in Fig. 4(b), showing incremental ethanol recovery as fermentation period increases.

Despite the operational issues encountered, it is evident that a fed-batch system with *ex-situ* hot microbubble stripping can increase ethanol production by *P. thermoglucosidasius* compared to a simple batch fermentation without ethanol stripping, which would be subject to product inhibition. With tuning of the initial batch fermentation time and fed-batch sugar addition rate, errors in which compromised the rate of metabolism of the organism in this study, a more complete coupled process could be undertaken that would identify the optimum ethanol and sugar steady state concentrations of the coupled processes.

**Table 1:** Ethanol yields from mass balance after 28 h of fermentation

Glucose in the fermentation broth (g), 4.57 L* @ 28.0 h			**Ethanol yield expected @ 28 h (90% theoretical)			Ethanol recovered (g) (87.16 g = 51% of the expected)				
total added (t = 0 h)	unused	consumed	total ethanol (g)	ethanol %(v/v)	ethanol (g/L)	condenser (bottle A)	vapour trap (bottle B, 200 mL)	vapour trap (500 mL)	vapour trap (15 mL)	remaining in the broth
583	212	371	170	4.7	37.2	22.2	1.0	3.6	0.32	60.0

* Final volume of reactor after 28 h (total of starting batch, fed-batch and batch additions)

** Ethanol yields are assumed to be around 90% of the theoretical maximum yield

### Ethanol mass balance

Figure 4(b) shows the ethanol concentration of samples collected from the condenser system linked to the MSU and the trap collecting the vapour leaving the bioreactor at each time point over the 5 stages of fermentation. These data were collected to allow a mass balance of ethanol/substrate over each of the stages and the entire process. The ethanol concentration in the vapour traps linked to the MSU condensate bottle and the bioreactor outlet were continuous measurements (i.e. the liquid in the trap was not changed during the experiment) and both the volume and ethanol concentration increased throughout the run.

A relatively slow increase in the volume of the post-MSU condensate liquid trap (30 mL over the entire experiment) demonstrated that the condenser system linked to the MSU captured most of the ethanol and water vapour (results not shown.) The losses from this vapour trap bottle were not measured as this was vented to the atmosphere, which may have reduced the total ethanol recovery figure. The trap placed on the gas outlet of the bioreactor increased by 50 ml over the course of the experiment with a final ethanol concentration of 6.6 g/L (Fig. 4b), giving an average increase of 1.8 mL/h. The smaller, subsequent trapping bottle increased from 15 mL to 19.5 mL. Figure 4b shows that a significant proportion of the ethanol in the post-bioreactor trap was generated in the last few hours of operation (stage V). During the fermentation period of 24–28 h, approx. 8.5 g of ethanol was collected by the condenser (bottle A) and the subsequent 200 mL trap (bottle B), while approx. 2.5 g of ethanol was recovered by the 500 mL and the 15 mL traps connected to the vapour exiting the bioreactor. Although the total volumes recovered from the post-bioreactor trap were small compared to the MSU condensate bottle, it should be noted that the bioreactor gas stream exited via a standard bioreactor condenser cooled by a small chiller unit, which would preferentially recondense water vapour together with some, but not all of the ethanol vapour [33]. So the efficiency of this reflux system was relatively poor under conditions of high productivity, and the selective loss of ethanol compared to the liquid phase concentration in the bioreactor, is evident.

Together with the ethanol recovered in the trap after the MSU condensers, that in the post-bioreactor traps can be used to calculate the total ethanol recovered during the experiment. From the sugar mass balance, it has been shown that 170 g (4.72% v/v) of ethanol could have been produced (Table 1) with only 51% of this ethanol accounted for, representing an apparent ethanol loss of 49%. From the problems encountered at the end of the batch phase and start of the fed-batch phase of the fermentation, we know that some of these “losses” are due to aerobic growth (with no ethanol production) in the recovery phase. Additionally, the use of micro-aerophilic, rather than fully anaerobic conditions will allow a small amount of respiration even under fermentative conditions. However, actual ethanol losses are not unexpected, as the concentrations in the liquid traps were high, relative to that in the bioreactor, and the smell of ethanol was evident in the fermentation room. Some ethanol losses during sampling, weighing/measuring and transferring to the collation bottle are also to be expected, in addition to the fact that the final trap bottles were gassed out to the atmosphere. Over the extended time of the experiment, these combined losses will be significant.

### Driving force for mass transfer

The calculated average ethanol generation rates with operating parameters can be used to estimate the average ethanol concentration in the MSU (not measured), based on our previous work [27]. The design equation is,

1 $${V}_{F}{\widehat{P}}_{E}={R}_{cir}\left({C}_{F}-{C}_{MSU}\right)$$

where ${V}_{F}$ is the fermenter volume, ${\widehat{P}}_{E}$ is the ethanol productivity, ${R}_{cir}$ is the liquid circulation rate between fermenter and the MSU, and ${C}_{F}$ and ${C}_{MSU}$ are the ethanol concentrations in the fermenter and MSU respectively. The average ethanol concentrations calculated using Eq. (1) are shown in Table 2.

**Table 2:** Comparison of ethanol concentration in the bioreactor (measured) and MSU (estimated)

Stage	${\varvec{C}}_{\varvec{F}}$ (g/L, measured)	${\varvec{C}}_{\varvec{M}\varvec{S}\varvec{U}}$ (g/L, estimated)
Stage II	7.27	7.10
Stage III	7.10	6.83
Stage IV	10.45	10.24
Stage V	14.80	13.95

Table 2 shows that the average ethanol concentrations in the bioreactor and MSU were close at all times. Note that Eq. (1) is designed for steady-state operation and these estimates are averages over the entire stage rather than instantaneous values. This demonstrates that the circulation rate set between the two vessels was sufficient to maintain a concentration inside the MSU driving rapid mass transfer.

Moving forward, even though we have been able to demonstrate ethanol production above the minimum economic levels of 4% v/v suggested by Lynd et al [8] with the aid of microbubble extraction, the repeatability and robustness of these results should be investigated in a manner where large data gaps are not present to allow for the analysis of what caused the bottleneck in the process. Some tuning should also be undertaken, as the ethanol concentration in the bioreactor for large parts of the investigation (up to 26 h) was lower than the optimum of 1.6% (v/v, 12.3 g/L). Additionally, the sugar addition rate should be reduced in line with the glucose consumption rate or continually adjusted using a control system to remain under the toxicity level. Finally, while this process is beneficial in that it produced ethanol from a waste product (waste-bread), the effects of using pretreated lignocellulose should be investigated and optimised as necessary.

## Conclusions

Hot microbubble gas stripping *ex-situ* has been demonstrated to be a feasible solution to the detrimental effects of product inhibition on the ethanol generation rate of the thermophile, *Parageobacillus thermoglucosidasius* strain TM333. This paper demonstrates microbubble extraction of ethanol using various operational modes, switching from batch to fed-batch (single batch medium additions and constant feeding rate of sugar medium), with both ethanol production and growth measurements (OD_600_) indicating that the use of microbubbles has no detrimental effects on growth and ethanol production by the production organism. Based on our previous studies [20], this work should be immediately applicable to the direct fermentation of starch present in waste bread. Furthermore, as *Parageobacillus* spp are known to be capable of metabolising almost all carbohydrate types found in lignocellulosic biomass, a major economic barrier to second generation biofuel production using thermophiles can be overcome. The maintenance of low ethanol concentrations in the fermentation vessel allows for continued growth and ethanol production by an organism that does not tolerate > 2% v/v ethanol, with the extraction of concentrated ethanol into vessels that could be cheaper to distil. Although some instability in the culture was experienced in the intermediate stages of the experiment, it is evident from the final few hours of fed-batch fermentation that with improved control of conditions very high productivity could be achieved. Indeed, for periods in the experiment, the ethanol generation was competitive with examples from the literature [31, 32]. This suggests that the stripping process used could be a valuable technology for the removal of bioethanol from fermentation processes to allow for fed-batch or fully continuous operation, which maintain long periods of high productivity by avoiding the periods of low productivity associated with repeated batch operations. Additionally, some indications of high-quality separations have been demonstrated where the condensate concentrations collected were found to be more concentrated than what would be produced using the traditional microorganism, *Saccharomyces cerevisiae*, using 1st generation substrates. In an industrial application of this process it would be preferable to introduce the ethanol-rich vapour from the MSU directly into a distillation column. This is a positive step towards a more secure energy future for transport applications, where biofuels are likely to continue to play a role for the foreseeable future.

## Data Availability

All data generated or analysed during this study are included in this published article. *Parageobacilllus thermoglucosidasius* TM333 is the property of ReBio Ltd.
